# Bioinformatic Analysis of *Chlamydia trachomatis* Polymorphic Membrane Proteins PmpE, PmpF, PmpG and PmpH as Potential Vaccine Antigens

**DOI:** 10.1371/journal.pone.0131695

**Published:** 2015-07-01

**Authors:** Alexandra Nunes, João P. Gomes, Karuna P. Karunakaran, Robert C. Brunham

**Affiliations:** 1 Bioinformatics Unit, Department of Infectious Diseases, National Institute of Health, Lisbon, Portugal; 2 Vaccine Research Laboratory, University of British Columbia Centre for Disease Control, Vancouver, Canada; University of the Pacific, UNITED STATES

## Abstract

*Chlamydia trachomatis* is the most important infectious cause of infertility in women with important implications in public health and for which a vaccine is urgently needed. Recent immunoproteomic vaccine studies found that four polymorphic membrane proteins (PmpE, PmpF, PmpG and PmpH) are immunodominant, recognized by various MHC class II haplotypes and protective in mouse models. In the present study, we aimed to evaluate genetic and protein features of Pmps (focusing on the N-terminal 600 amino acids where MHC class II epitopes were mapped) in order to understand antigen variation that may emerge following vaccine induced immune selection. We used several bioinformatics platforms to study: *i)* Pmps’ phylogeny and genetic polymorphism; *ii)* the location and distribution of protein features (GGA(I, L)/FxxN motifs and cysteine residues) that may impact pathogen-host interactions and protein conformation; and *iii)* the existence of phase variation mechanisms that may impact Pmps’ expression. We used a well-characterized collection of 53 fully-sequenced strains that represent the *C*. *trachomatis* serovars associated with the three disease groups: ocular (N=8), epithelial-genital (N=25) and lymphogranuloma venereum (LGV) (N=20). We observed that PmpF and PmpE are highly polymorphic between LGV and epithelial-genital strains, and also within populations of the latter. We also found heterogeneous representation among strains for GGA(I, L)/FxxN motifs and cysteine residues, suggesting possible alterations in adhesion properties, tissue specificity and immunogenicity. PmpG and, to a lesser extent, PmpH revealed low polymorphism and high conservation of protein features among the genital strains (including the LGV group). Uniquely among the four Pmps, *pmpG* has regulatory sequences suggestive of phase variation. In aggregate, the results suggest that PmpG may be the lead vaccine candidate because of sequence conservation but may need to be paired with another protective antigen (like PmpH) in order to prevent immune selection of phase variants.

## Introduction

*Chlamydia trachomatis* is an obligate intracellular human bacterial pathogen, comprised of 15 to 18 major serovars. Serovars A-C are responsible for ocular infections that result in trachoma [1]. Serovars D-K cause sexually transmitted diseases such as cervicitis and pelvic inflammatory disease (PID), and globally are an important infectious cause of infertility and ectopic pregnancy in women [2]. Serovars L1-L3 also enter through the ano-urogenital tract, but may dissiminate via infection of macrophages to regional draining lymph nodes, causing lymphogranuloma venereum (LGV) [3]. *C*. *trachomatis* is the major bacterial cause of sexually transmitted infections (STIs), accounting for ~106 million of the 500 million new cases of STIs that occur each year worldwide [4]. In Europe, almost half of the estimated 47 million STI cases are due to *C*. *trachomatis* [4], while nearly 1.4 million infections are reported each year in the United States [5]. Although the estimated global economic burden is uncalculated, over $516 million are spent annually in direct medical costs on genital chlamydial infections in the United States, making *C*. *trachomatis* the most costly infection among the nonviral STIs [6–7].

Despite screening and treatment public health programs to control *Chlamydia*, the incidence of *C*. *trachomatis* infection has increased [4, 8–10]. Thus, there is an urgent need for an efficacious vaccine that prevents acquisition and transmission of infection and the development of pelvic inflammatory disease sequelae. Cumulative studies in animal models and human infections [8, 11–23] have shown that systemic and mucosal CD4 T cell-mediated immunity is necessary for protection against *C*. *trachomatis* infection. Among antigen candidates that have been studied [11, 15, 24], members of the polymorphic membrane protein family (PmpA-I) have shown promise as vaccine components as they are dominant antigenic targets for cellular immune responses [25–29]. Four of the nine Pmps (PmpE, PmpF, PmpG and PmpH) have been identified via immunoproteomics as dominant T-cell antigens with multiple MHC class II binding peptides for both *C*. *trachomatis* and *C*. *muridarum* and observed to be protective in the murine genital tract infection model [25, 28–30]. The fact that each Pmp generates different peptides recognized by different MHC class II haplotypes confers on them the capability of immunizing outbred populations [25]. In the murine model, a PmpG epitope was found to persist on splenic antigen presenting cells for at least 6 months [26]. Tetramer staining also demonstrated PmpG as one of the quantitatively dominant antigens recognized by murine CD4 T cells [17].

Pmps are *Chlamydia*-specific outer membrane proteins whose precise functions remain unknown, but which have been implicated in pathogenesis and host cell adherence. As typical type V autotransporters [31–32], Pmps are capable of translocating to the bacterial surface their N-terminal Sec-dependent leader sequence (passenger domain), containing multiple short repetitive motifs (GGA(I, L, V) and FxxN) [33]. The proteins may also undergo complex infection-dependent post-translational proteolytic processing [34–37]. These proteins mediate *in vitro* chlamydial attachment to human epithelial and endothelial cells [34, 38]. Previous bioinformatics analyses suggested that six of the 9 *pmp*s are under positive selection either driving bacterial adaptation to specific niches (ocular conjunctiva, epithelial-genitalia and lymph nodes), or during a strains’ diversification within a particular niche [39]. Numerous mutations within bioinformatically predicted HLA class I and II T-cell epitopes for the N-terminal domain for one Pmp (PmpF) have already been identified [40]. Pmps were also found in tissue culture to be variably expressed at the chlamydial cell surface, where each protein seemed subjected to an independent high-frequency on/off switching at the inclusion level [41].

Therefore we aimed to bioinformatically assess the allelic and phase variation of these antigens, in particular for the four most promising vaccine candidates PmpE, PmpF, PmpG and PmpH, in order to understand if putative antigenic escape variants may emerge following vaccination. We performed a detailed bioinformatic analysis using a well-characterized collection of 53 strains whose genomes have been fully sequenced [42–43] and which represent *C*. *trachomatis* serovars associated with the three canonical disease groups: ocular, epithelial-genital and LGV. By encompassing strains from temporally and geographically diverse sources around the world, this collection should capture *C*. *trachomatis* genetic diversity. Overall, we analyzed 25 strains from epithelial-genital serovars, 20 strains from LGV serovars, and eight strains from ocular serovars.

## Materials and Methods

### Genetic and phylogenetic analyses

In order to assess the genetic variability of PmpE, PmpF, PmpG and PmpH within *C*. *trachomatis*, 53 genome sequences representative of distinct or same-serovar strains were retrieved from GenBank and aligned using the progressiveMauve algorithm of Mauve 2.3.1 [44] with the default parameters and an initial match seed weight appropriate for 1MB genomes. Basically, Progressive Mauve performs a recursive anchor search and a full gapped anchored alignment of the genome sequences using a modified MUSCLE algorithm. For all *pmp*s, individual alignments were extracted from the whole-genome alignment, and visually inspected with MEGA6 software (http://www.megasoftware.net) for further correction.

For each *pmp*, MEGA6 was also used to estimate the number of gene variable sites and to compute overall mean distances and matrices of pairwise comparisons at both nucleotide and protein level, based on the number of differences and *p*-distance value (that calibrates the obtained differences relative to the total number of sites under comparison) among the 53 strains, along with the respective standard error estimates (bootstrap = 1000). Evaluation of variable sites and mean genetic distances was also performed within and between the three disease groups. We also evaluated the impact of the genetic heterogeneity of each group of strains in the overall polymorphism of each *pmp*, through a sliding-window analysis using the DNA polymorphism tool of the DnaSP software, version 5 [45], with a window size and step size of 15.

Phylogenetic relationships among strains were inferred with MEGA 6, by using the Neighbor-Joining method [46] in conjunction with a bootstrap re-sampling strategy (1000 replicates), as previously described [47]. Evolutionary nucleotide distances were estimated with the Kimura 2-parameter (K2P) model [48] that takes into account transitional and transversional substitution rates, while assuming identical nucleotide frequencies and invariable substitution rates among sites [49]. At the protein level, evolutionary distances were computed based on the number of differences. Because of the different lengths of the sequences, the pairwise-deletion option was chosen to remove all sites containing missing data or alignment gaps from all distance estimations, only when the need arose and not prior to the analysis.

In order to identify specific regions that most contribute to the phylogenetic segregation of taxa within each Pmp, we evaluated the similarity both among the 53 strains and between disease groups. Briefly, SimPlot 3.5.1 software [50] was used to plot a codon-based nucleotide similarity score of each strain against a particular query by estimating pairwise distances with K2P model, without excluding gaps among sequences and considering a transition-transversion substitution rate of 2. The similarity estimations were performed in a sliding window size that ranged from 40 bp for *pmpG* to 80 bp for *pmpF* (adjusted according to polymorphism degree), moved across the alignment in a step size of 3 bp. In parallel, SWAAP 1.0.3 software [51] was used to compute the percentage of amino acid (a.a) identity among each pair of sequences throughout all Pmps, over a sliding window that ranged from 20 a.a for both PmpG and PmpH to 40 a.a for PmpF, and a step size of 3 a.a.

These analyses were also performed using gene segments encoding the first ~600 amino acids of each Pmp, as they are part of the surface exposed passenger domain where all MHC class II binding peptides were experimentally mapped [25] (see Results and Discussion section).

### Analysis of protein features

Pmps are predicted to have adhesin functions [34, 38, 52]. By using the SeqBuilder module of LaserGene (DNASTAR) we therefore assessed the differential presence among all 53 strains of the repetitive GGA(I, L, V) and FxxN motifs as well as of cysteine residues in each Pmp. Moreover, to shed light on the putative impact of Pmps’ variability on surface probability and immunogenicity, we used the Protean program (DNASTAR) to perform a comparative analysis of the protein sequences. While the surface probability parameter predicts the likelihood of a given region lying on the surface of a protein using the approach of Emini *et al*. [53], the analysis of immunogenicity uses the approach of Jameson and Wolf [54] that combines several methods for protein structural features (like hydrophilicity, surface probability, flexibility and secondary structure) to predict potential antigenic determinants. For both analyses, the default parameters of each method were used, with surface regions predicted by forming the product of residue specific surface propensities over a range of 5 amino acids and based on a surface decision threshold >1. In order to facilitate the latter analyses, we used one strain representative of the main branches of the respective tree generated with the 53 fully-sequenced genomes, as these strains accurately represent the Pmp genetic backbone of the remainder same-branch taxa. The ocular strain A/Har13, the two epithelial-genital strains D/UW3 and E/150, and the LGV strain L2b/UCH1 were selected as they were among the strains found to be commonly present in the different main branches of all phylogenetic trees. Considering that some Pmps have an additional divergent branch containing genital strains, we opted for including an extra epithelial-genital strain to increase the confidence of the analysis. Thus, the strain G/11074 was also used for the analysis of both PmpH and PmpG, while the strains G/11222 and E/SW2 were used for the analysis of PmpF and PmpE, respectively. After performing preliminary data, we observed that including other strains besides these ones, did not alter the final output. In order to increase the probability of observing relevant disparities among strains for surface probability and immunogenicity, we specially focused on regions falling below a similarity cut-off of 85% (based on SWAAP plots).

### Analysis of phase variation

Analysis of phase variation was performed by using different approaches by considering several genetic features known to underlie phase variation mechanisms, such as short sequence repeats (homopolymeric and non-homopolymeric tracts), IS-like sequences, frameshift mutations, small indels, hairpin structures, RNase E cleavage sites, and promoter sequences [55–57]. First, we checked the differential presence of homopolymeric tracts and small indels among all 53 strains by using SeqBuilder (DNASTAR). For each operon, *in silico* promoter predictions were made by using both the Neural Network Promoter Prediction (NNPP, http://www.fruitfly.org/seq_tools/promoter.html) and the BPROM software (Softberry, http://linux1.softberry.com/berry.phtml?topic=bprom&group=programs&subgroup=gfindb), to find elements resembling putative σ^66^, σ^28^ and/or σ^54^ promoter sequences that may be differentially present among strains, and thus used as fine-tune transcriptional *pmp* regulators. Moreover, several putative regulatory elements that are known to affect transcription or translation were also searched throughout each *pmp* operon and associated regulatory regions using SeqBuilder (DNASTAR). Briefly, the differential existence of putative consensus cleavage sites for RNase E was examined among strains [58–60]. RNaseE is the major endonuclease that generally initiates mRNA degradation in most bacteria [61]. We also searched for the presence of putative Shine-Dalgarno ribosome binding sequences (RBS) [62] and previously described chlamydial RBS [63–66].

## Results and Discussion

### Global polymorphism analysis

In view of *Chlamydiae*’s reductive evolution, it is remarkable that the *pmp*s vary in number across species and encompass a sizeable chromosome portion (3–5%) [67]. For instance, over 13% of the *C*. *trachomatis* specific coding capacity is restricted to this gene family [68]. The evaluation of the mean genetic distances among all 53 strains revealed that *pmpF* was the most polymorphic *pmp* gene exhibiting the highest mean nucleotide substitution (214.3 (SE 8.9)) and also the highest *p*-distance value (0.0691 (SE 0.0029)), which corresponded to a mean of 72.4 (SE 5.0) (7.0%) amino acid substitutions. This degree of polymorphism is followed by *pmpH* and *pmpE* with an overall nucleotide variability of 3.6% and 2.4%, respectively. *pmpG* was observed to be the least variable gene with a polymorphism 6.9-fold lower than that of *pmpF*. In fact, *pmpG* exhibited a mean of nucleotide substitutions among all 53 strains of 31.1 (SE 3.7) (1.0%), corresponding to a mean of 13.5 (SE 2.6) (1.3%) amino acid alterations. The high variability shown by some Pmps accords with a recent analysis encompassing ~98% of the *C*. *trachomatis* core genome [69] which placed *pmpF*, *pmpE* and *pmpH* within the top 20 most polymorphic chromosomal genes. The set of polymorphic genes includes other important antigens-coding genes, such as CT681/*ompA* and the paralogously related *pmp*s CT049-CT051 [70–72]. Considering Pmps’ outer membrane localization and putative dual function in adhesion and pathogenesis, the high polymorphism is expected to promote multiple antigenic and/or adherence phenotypes that may influence strains’ pathogenic diversity and tissue specificity.

**Fig 1:**
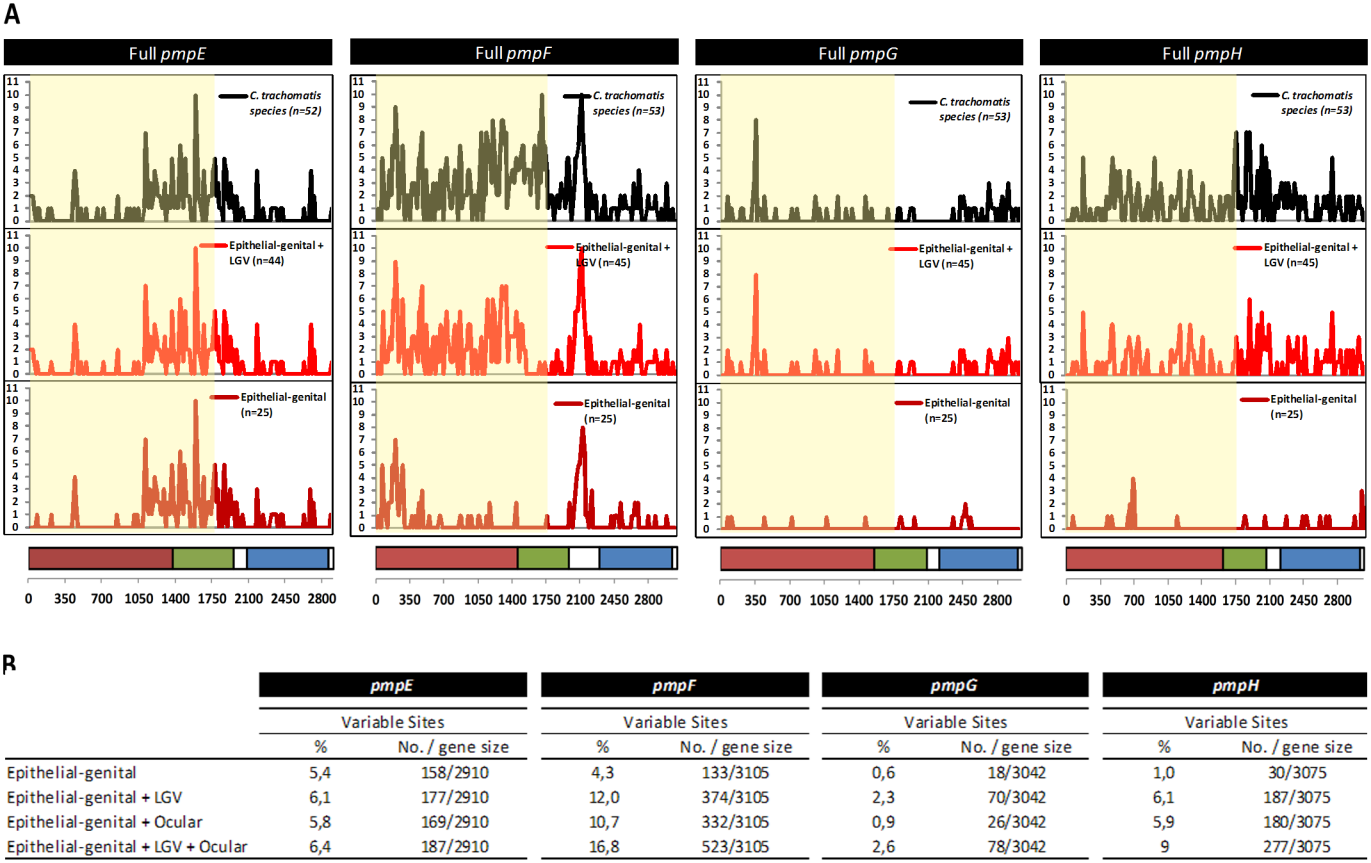
Global polymorphism analysis. A) Sliding-window analysis of the genetic variability throughout each *pmp* (window and step size of 15 bp). For each *pmp*, the black plot represents the polymorphism among all strain collection, while the remainder plots represent the impact of removing strains from the ocular group (red plot) and from the LGV group (magenta plot) on the global polymorphism. The region highlighted in yellow encompasses the 1^st^ 600 amino acids (used in vaccine attempts) where further analyses were performed. The horizontal bars below the plots represent the typical domains of these autotransporter proteins: passenger domain (magenta), middle domain (green), and C-terminal autotransporter domain (blue). B) Impact in both the number and percentage of variable sites found among *C*. *trachomatis* strains after the successive addition of LGV and ocular strains to the epithelial-genital group.

By performing a polymorphism sliding-window analysis throughout each gene (Fig 1), we observed that most of the genetic variability in *pmpE* is concentrated into the mid-region of the gene (~1050–2100 bp), while a greater nucleotide substitution density is seen within the first two-thirds of *pmpF*. In contrast, for both *pmpG* and *pmpH*, the overall variability appears to be homogeneously distributed throughout each gene, although a polymorphism peak is seen around position 350 for *pmpG* (corresponding to three amino acids that are specific of LGV strains) We also found distinct scenarios among the four *pmp*s by evaluating the contribution of the different disease groups to the overall species polymorphism (Fig 1). For both *pmpF* and *pmpH*, the three disease groups seem to contribute to *C*. *trachomatis* diversity, as seen by the successive increase in the overall genetic variability after the independent addition of either LGV or ocular strains to the group of epithelial-genital strains. In fact, the number of variant sites found among epithelial-genital strains augmented ~3-fold for *pmpF* and ~6-fold for *pmpH* with addition of LGV strains, and an increase of 1.5-fold was seen after the subsequent inclusion of ocular strains for both genes. For *pmpE*, the overall species polymorphism is essentially provided by the genetic heterogeneity among the epithelial-genital strains, which harbor almost 85% of the total 187 variant sites found. The dissimilar mutational pattern observed for each *pmp* may imply differences in how each Pmp fulfills adhesive and antigenic functions in specific niches. Distinct regions were previously identified within *pmpE*, *pmpF* and *pmpH*, where clusters of mutations were found, and were associated with strains’ clustering by cell-tropism or ecological success [73]. In support of this notion, these four *pmp*s were found to be differently targeted by positive selection in each niche, being potentially involved in one or more adaptive processes [39]. For instance, based on bioinformatic predictions that rely essentially on the distribution and exclusive character of nonsilent changes, *pmpF* is identified to encode specific cell tropism to both the ocular epithelium and mononuclear phagocytes. Besides niche-specific adaptation, some of the *pmp*s were also predicted to evolutionarily impact strains’ niche-specific pathogenicity, as the case of *pmpG* and *pmpH*, which appear to contribute to the pathogenic diversity among LGV-causing strains [39]. Both *pmpG* and *pmpH* have over-accumulated nonsynonymous substitutions (about 4-fold more frequent than silent mutations) that result in protein variation among LGV strains [74]. Multiple factors may contribute to the genetic diversity of *pmp*s, such as recombinational hotspots involving this large family of paralogous genes since multiple recombination hotspots have been identified throughout the genome [43, 75]. Overall, these data suggest the existence of variant tissue-specific host-interaction motifs that involve different Pmps.

### Genetic and phylogenetic analyses of Pmps’ N-terminal 600 amino acid domains

Previous immunoproteomic studies using the murine genital infection model [25] revealed that PmpE, PmpF, PmpG and PmpH contain different MHC class II binding peptides in the N-terminal half for both *C*. *muridarum* and *C*. *trachomatis* serovar D with between one and three different peptides *per* MHC allele. Therefore, after an initial genetic analysis of the overall polymorphism for the entire genes, the subsequent analyses (genetic, phylogenetic, and study of protein features) were restricted to gene segments encoding the 1^st^ ~600 amino acids of each Pmp that encompass both the surface exposed passenger and middle domains (Fig 1) where all MHC class II-bound peptides were experimentally mapped [25]. The selected gene segments harbor almost 75% of all variant sites estimated for the whole *pmpF* and *pmpE* genes and at least 50% of the variant sites for *pmpG* and *pmpH*.

The phylogenetic reconstructions based on the first 600 amino acids of each Pmp (S1 Fig), mirror the tree topologies for the whole proteins (S2 Fig) and support the correlation between *pmp* polymorphism and a strains’ phenotypic association with disease state. For instance, a perfect segregation of strains by full-tropism was observed for PmpH, with strains representative of the three disease groups appearing clustered in distinct separated clades. For all Pmps, the analysis of the matrices of pairwise distances revealed a low heterogeneity within both ocular and LGV strains (Table 1). In contrast, the average evolutionary divergence among the epithelial-genital group varies among Pmps, with PmpE and PmpF exhibiting the highest divergence, 14.5 a.a (SE 2.0) and 14.0 (SE 2.3), respectively, while PmpG and PmpH are much less polymorphic, displaying a polymorphism 7- to 9-fold lower. However, if LGV strains are included in a global group of genital strains, the average amino acid distances increases as epithelial-genital strains are highly dissimilar from LGV strains for some Pmps (Table 1). Overall, these results show that the 1^st^ 600 a.a of PmpG is highly conserved in this 53 strain collection, even among all genital strains (epithelial-genital and LGV), for which the observed mean amino acid distances are about 2-, 3- and 7-fold lower than those seen for PmpH, PmpE and PmpF, respectively. Of note, the segment of the 1^st^ 600 a.a of PmpH is also highly conserved if one considers solely epithelial-genital strains, where no more than 2 a.a differences are seen.

**Table 1:** Mean amino acid distances within the 1^st^ 600 amino acids of each Pmp.

	PmpE		PmpF		PmpG		PmpH	
	No. diff.	SE	No. diff.	SE	No. diff.	SE	No. diff.	SE
Overall mean	28,0	3,4	53,2	4,4	7,7	1,8	20,9	2,7
*Within Groups*								
Ocular	1,9	0,8	1,0	0,5	0,8	0,4	0,0	0,0
Epithelial-genital	14,5	2,0	14,0	2,3	1,7	0,9	2,0	0,8
LGV	0,0	0,0	0,0	0,0	0,6	0,3	0,8	0,4
Genital (with LGV)	19,5	2,5	52,2	4,7	7,4	1,8	16,9	2,7
*Between Groups*								
Ocular / Epithelial-genital	39,2	5,3	65,5	6,9	3,4	1,4	29,9	5,0
Ocular / LGV	47,4	6,3	111,0	8,9	11,6	3,1	45,5	6,3
Epithelial-genital / LGV	19,0	3,1	84,0	7,8	12,0	3,2	31,0	5,0

### Analysis of peptide features

**Fig 2:**
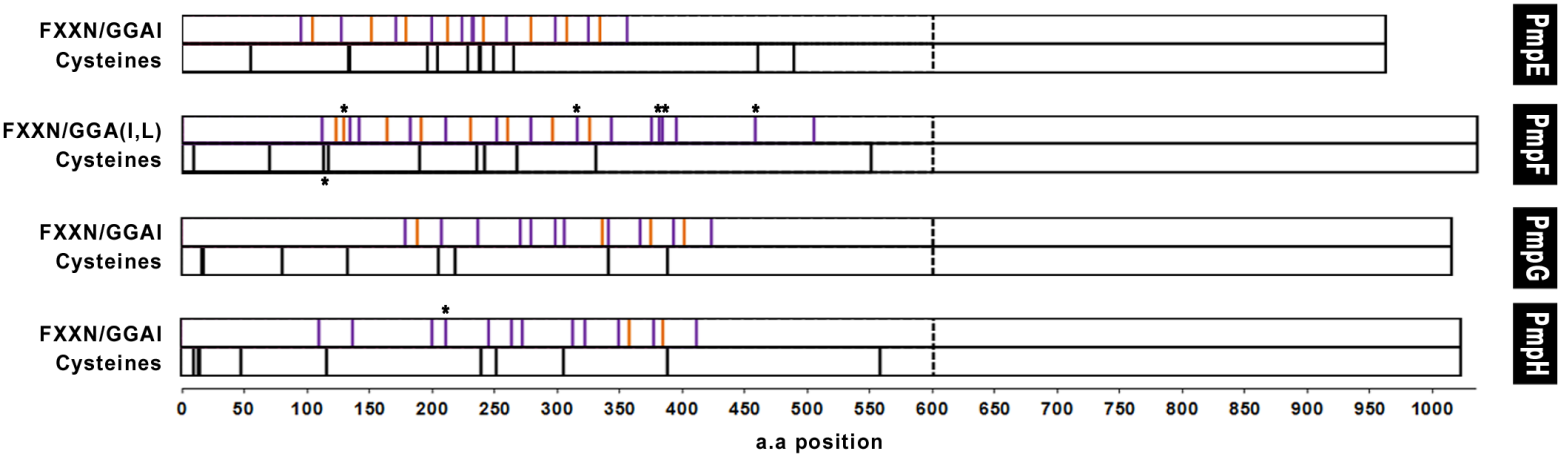
Distribution of FxxN motifs (purple), GGA(I, L) motifs (orange), and cysteine residues (black) within the 1^st^ 600 a.a (limited by the vertical dashed line) of each Pmp. Asterisks indicate motifs nonconserved among all 53 strains.

**Fig 3:**
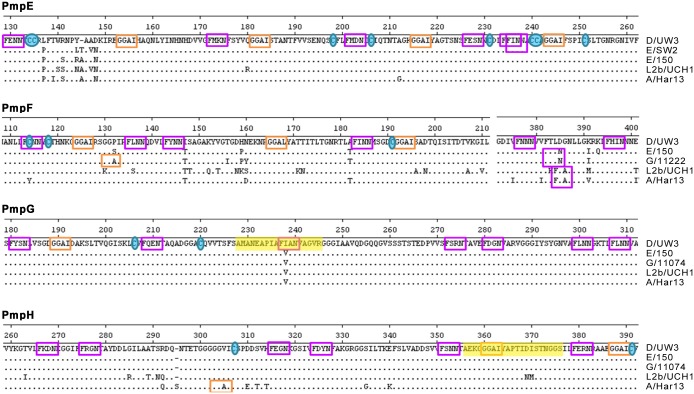
Pmp alignments showing examples of protein regions containing high concentration of FxxN motifs (purple), GGA(I, L) motifs (orange), and cysteine residues (blue). MHC class II (I-Ab)-bound *C*. *trachomatis* peptides are highlighted in yellow.

A hallmark of the chlamydial Pmp family is the presence of multiple repeats of the tetrapeptide motifs GGA(I, L, V) and FxxN in their N-terminal half. The repetition of these motifs is seen in very few non-chlamydial proteins, and has been suggested to be directly involved in adherence processes [33, 76]. Thus, considering that Pmps likely have adhesive functions [34, 38] and were found to be variably expressed at the chlamydial surface *in vitro* [41], we mapped the location and determined the number of the GGA(I, L, V) and FxxN as well as of the cysteine residues within the 1^st^ 600 a.a of each Pmp for all 53 strains (Fig 2). We observed that the FxxN motif occurs on average 12.3-times *per* Pmp, being ~2-fold more frequent than GGAI motifs, which appear on average 5.8-times *per* Pmp. The mean incidence of FxxN in the remaining *C*. *trachomatis* proteome was shown to be 0.73 *per* protein, whereas GGAI is predicted to be present as single copy in only 10 other *Chlamydia* proteins [33]. No GGA(I, L, V) or FxxN motif is found outside the 1^st^ 600 a.a for each Pmp, which is consistent with the exclusive presence of adhesion domains in protein regions that interact with the host. The number of conserved GGAIs varied among the four Pmps, ranging from two for PmpH to eight for PmpE. All Pmps displayed 11 conserved FxxN motifs; one of these fell within the MHC class II (I-A^b^)-bound *C*. *trachomatis* serovar D-derived peptide (AMANEAPIAFIANVAG) (Fig 3) recently identified in PmpG [25].

The distribution of the two types of peptide motifs is nonrandom, being mainly clustered within a discrete region that spans between ~220 a.a for PmpH to ~280 a.a for PmpF (Fig 2). Most of these motifs appear as doublets of FxxN-x_2-25_-FxxN and FxxN-x_4-20_-GGAI, whose differential occurrence, number and spacing vary both among Pmps and among different groups of strains for the same Pmp. They were also found to be in close proximity or adjacent to cysteine residues. The number of conserved cysteines ranges from eight for PmpG to 12 for PmpE. Considering that cysteines are known to play an important role in maintaining structural motifs, such GGAI/FxxN nonrandom distribution and possible cooperative action with the cysteine residues may yield unique structural or functional constraints for interaction with the host cell.

We also observed that the majority of the tetrapeptide motifs occurred in close proximity or fall within regions containing SNPs differentiating specific groups of strains (Fig 3), which may widen adhesion or immunogenic differences within each Pmp, as described for the major chlamydial membrane protein MOMP [77–79]. In support of this, a typical conserved FxxN-X_5_-GGAI doublet was found to overlap the MHC class II (I-A^b^)-bound peptide (AEKGGGAIYAPTIDISTNGGS) identified in PmpH [25], which is conserved solely among ocular and epithelial-genital strains (Fig 3). This epitope is also in very close proximity to another conserved FxxN-X_4_-GGAI doublet and a conserved cysteine residue. Based on bioinformatic predictions, none of the three MHC class II (I-Ab)-bound *C*. *trachomatis* serovar D-derived peptides recently identified in PmpE, PmpG and PmpH [25] fall in regions that impact variability in immunogenicity and surface exposure of the respective proteins (S3 Fig). Although the precise function of the tetrapeptide motifs is yet unknown, it has been shown by using deletion analysis that Pmps’ adhesion capability requires at least the presence of one of these doublets [80]. Also, each Pmp was shown to exhibit *in vitro* a distinctive adhesion profile depending on the human cell type (epithelial *versus* endothelial) [34]. The mutational pattern of PmpE, PmpF, PmpG and PmpH was shown to be associated with high efficiency of *in vitro* attachment to host cells [81]. It has been hypothesized that these short repetitive motifs may be involved in maintaining the Pmp conformation that promotes adhesion, and/or might directly mediate interaction with human receptors [80]. Therefore, the wide variation of these repetitive motifs seen among intra- and inter-Pmps (Figs 2 and 3), is consistent with a role in the formation of niche-specific binding “receptors” for host-interaction.

### Analysis of phase variation

Since *pmp*s have been suggested to undergo phase variation-like mechanisms to promote multiple antigenic and/or adherence phenotypes [41], we searched for putative phase variation features on each gene [55–57]. In distinction to *pmpE*, *pmpF* and *pmpH* that reveal no poly(C) or poly(G) stretches, *pmpG* exhibits an in-frame poly(G) tract of nine residues for all non-LGV strains and eight residues for LGV strains (data not shown). This mirrors that found for *C*. *pneumoniae pmpG* family [33, 82–84], suggesting that slipped-strand phase variation [55, 57] may be a common phenotype for PmpG. However, it is not known if the observed poly(G) tract influences *pmpG* expression in *C*. *trachomatis*. Although all annotated genome sequences appear to possess a functional PmpG, we cannot be certain that *C*. *trachomatis* strains only harbor in-frame poly(G) tract in *pmpG*. In fact, it is known that this type of regions may yield biased data in some Next Generation Sequencing. So, it is possible that strains with dissimilar number of “G” (or even a mixture of clones showing different number of “G”) have not been properly annotated. Nevertheless, as only PmpG shows a poly-G tract, which may be involved in phase variation, and because its sequence is highly conserved among all genital strains (including LGV strains), this may be its major mechanism of antigen or niche variation. We also found a non-homopolymeric repetitive motif “PAPAPAPA” in PmpH for some epithelial-genital strains, which is shorter for other epithelial-genital strains and absent for both the LGV and ocular groups (data not shown). A Blast search for this motif was not informative about its putative role, as it seems to be commonly present in diverse proteins, such as regulatory proteins, acid-shock proteins and acetyl-CoA carboxylases, from different species. Hypothetically this genetic feature could be associated with phase variation and would exclusively impact PmpH immunogenicity of epithelial-genital strains. Although this hypothesis is speculative, if PmpH is used as a vaccine component, the use of peptides encompassing all “PA” combinations may be prudent.

It is known that the insertion-excision of mobile elements (like IS-elements) may also impact gene expression [55], and the presence of putative remnants of IS-like elements flanked by direct target repeats has already been described for both *pmpB* and *pmpC* [73]. However, no such elements were found in the four *pmp*s under evaluation.

Past studies have shown that expression regulation of *C*. *trachomatis* genes is controlled at both transcriptional and translational levels, thus involving multiple complex aspects, like DNA supercoiling, heterogeneity within promoter sequences, cis- and trans-regulatory elements, and mRNA stability. It is known that various chlamydial genes are regulated by two or three different promoters, often with multiple σ factor binding (σ^66^, σ^28^ and/or σ^54^) [85]. We found putative σ^66^ promoters within both *pmpFE* and *pmpGH* regulatory regions by using *in silico* predictions (S1 and S2 Tables), but no elements resembling a σ^28^ or σ^54^ promoter sequence. In distinction to *pmpFE*, the most promoter predicted for the *pmpGH* operon contains a transcritptional start site (TSS) that has already been experimentally identified for the L2b/UCH1 strain [86], and it reveals a variable site in the -35 element that distinguishes LGV from epithelial-genital strains. Some variable nucleotide sites are also seen near the -10 elements, the TSSs, or A/T spacer, and several putative consensus RNase E cleavage sites were found throughout both operons and their regulatory regions. The polymorphisms found close or inside Shine-Dalgarno regulatory elements and the nonconservation of RNase E cleavge sites, may suggest heterogeneous expression among strains for both *pmpFE* and *pmpGH* operons, supporting previous experimental findings showing Pmp expression differences not only between L2 and E strains, but also within same-serovar strains [87].

## Conclusions

Recent vaccine studies in the murine model [29, 88] provided evidence that a vaccine composed of PmpEFGH plus MOMP formulated with a Th1 polarizing adjuvant, was more immunogenic and cleared infection faster than a single antigen vaccine. The suitability of a Pmp-based vaccine will be influenced by antigenic variability displayed during infection of the genital tract. The *in silico* predictions from the present study suggest that PmpF and PmpE may be less reliable antigens for vaccine purposes due to high sequence polymorphism. This polymorphism, together with putative alterations in structural constraints provided by heterogenity among strains in GGA(I, L)/FxxN motifs and cysteine residues, also suggest a possible role in immunogenicity variability. By contrast, the low polymorphism and high conservation of protein features for PmpG and to a lesser extent for PmpH, suggest that these proteins may be better vaccine candidates. Since phase variation may impact PmpG expression a single-component PmpG subunit vaccine may not provide protection against infection due to phase variation. Based on bioinformatics analysis we suggest that pairing of PmpG with PmpH could be a viable approach, in order to provide a range of epitopes for CD4+ T cell recognition among different MHC genetic backgrounds and to provide cross-protection against multiple antigenic variants of *C*. *trachomatis*.

## Supporting Information

S1 Fig Phylogenetic trees of the 1st 600 amino acids of each Pmp. (PDF)

S2 Fig Phylogenetic trees of each Pmp. (PDF)

S3 Fig Amino acid (a.a) identity throughout the 1st 600 a.a of PmpE (panel A), PmpF (panel B), PmpG (panel C) and PmpH (panel D) among strains representative of the main branches of each phylogentic tree. (PDF)

S1 Table *In silico* promoter predictions for *pmpFE* operon regulatory region. (PDF)

S2 Table *In silico* promoter predictions for *pmpGH* operon regulatory region. (PDF)
