# Supplementary figures and images for: Bioinformatic Analysis of Chlamydia trachomatis Polymorphic Membrane Proteins PmpE, PmpF, PmpG and PmpH as Potential Vaccine Antigens
Source: PLoS One. 2015 Jul 1;10(7):e0131695. doi: 10.1371/journal.pone.0131695 (PMC4488443; doi:10.1371/journal.pone.0131695)

## PmpE

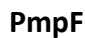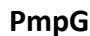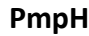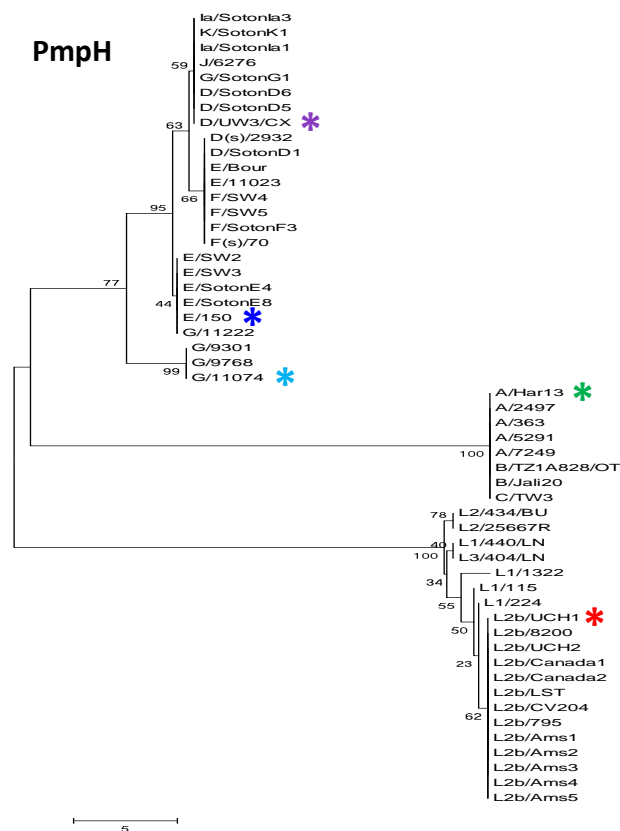

Supplement: S1 Fig — (PDF) [file pone.0131695.s001.pdf]
