# Supplementary material for: Bioinformatic Analysis of Chlamydia trachomatis Polymorphic Membrane Proteins PmpE, PmpF, PmpG and PmpH as Potential Vaccine Antigens
Source: PLoS One. 2015 Jul 1;10(7):e0131695. doi: 10.1371/journal.pone.0131695 (PMC4488443; doi:10.1371/journal.pone.0131695)

S2 Fig. Phylogenetic trees of each Pmp. Asterisks represent the strains that were chosen for comparative purposes for protein analyses.

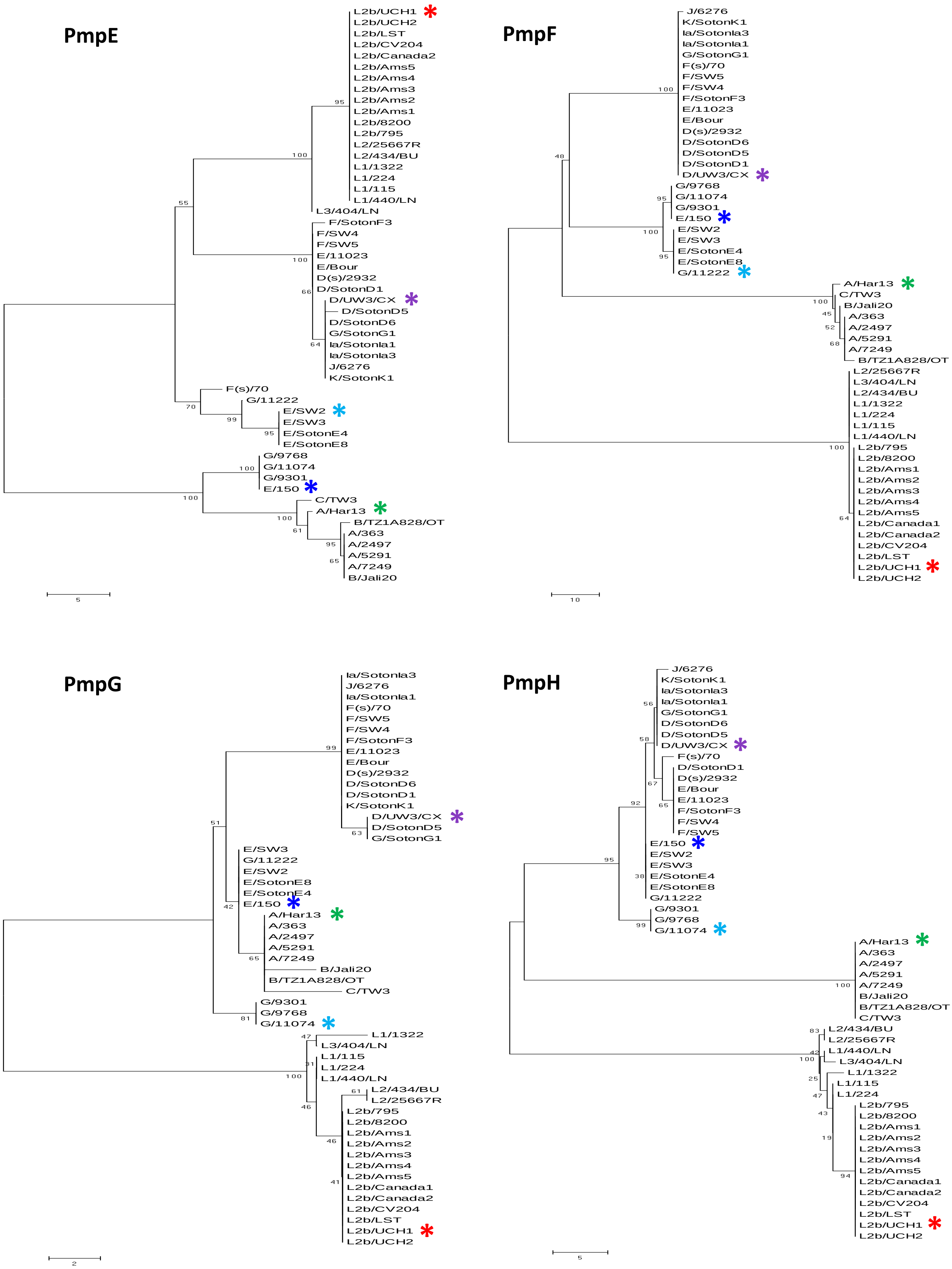

Supplement: S2 Fig — (PDF) [file pone.0131695.s002.pdf]
