# Supplementary material for: Bioinformatic Analysis of Chlamydia trachomatis Polymorphic Membrane Proteins PmpE, PmpF, PmpG and PmpH as Potential Vaccine Antigens
Source: PLoS One. 2015 Jul 1;10(7):e0131695. doi: 10.1371/journal.pone.0131695 (PMC4488443; doi:10.1371/journal.pone.0131695)
Supplement: S3 Fig — (PDF) [file pone.0131695.s003.pdf]

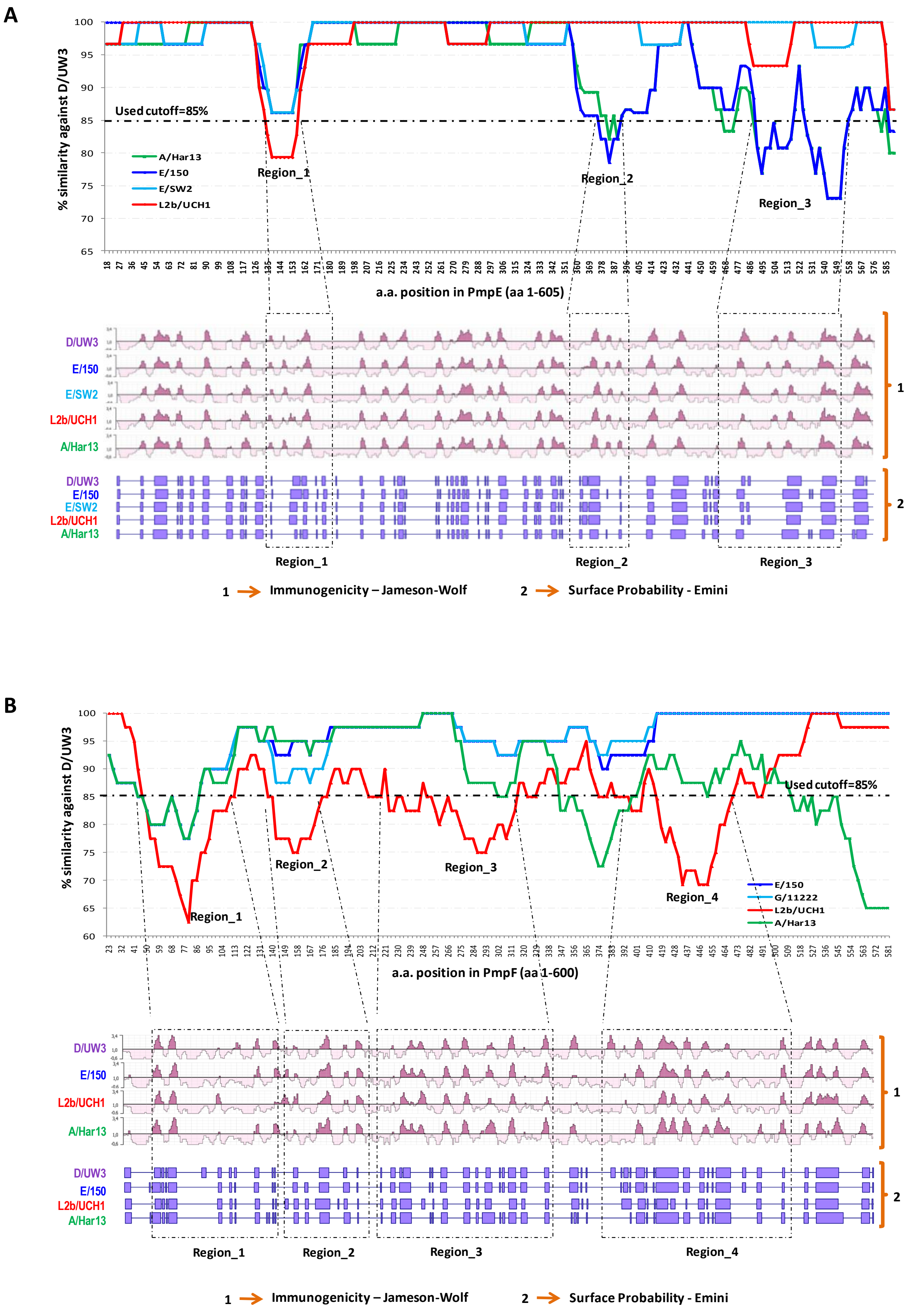

A
% similarity against D/UW3
Used cutoff=85%
A/Har13
E/150
E/SW2
L2b/UCH1
Region_1
Region_2
Region_3
a.a. position in PmpE (aa 1-605)
D/UW3
E/150
E/SW2
L2b/UCH1
A/Har13
1
2
Region_1
Region_2
Region_3
1 → Immunogenicity – Jameson-Wolf
2 → Surface Probability - Emini
B
% similarity against D/UW3
Used cutoff=85%
Region_1
Region_2
Region_3
Region_4
E/150
G/11222
L2b/UCH1
A/Har13
a.a. position in PmpF (aa 1-600)
D/UW3
E/150
L2b/UCH1
A/Har13
1
2
Region_1
Region_2
Region_3
Region_4
1 → Immunogenicity – Jameson-Wolf
2 → Surface Probability - Emini

C

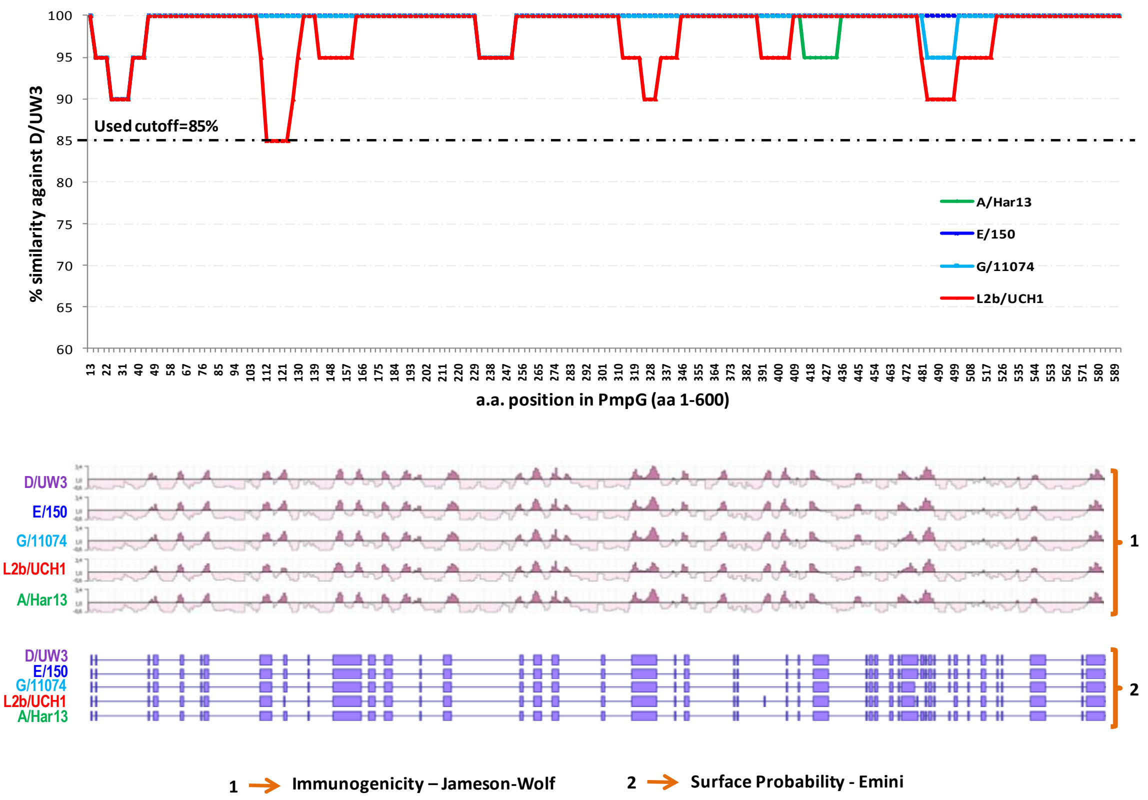

100
95
90
85
80
75
70
65
60
% similarity against D/UW3
Used cutoff=85%
A/Har13
E/150
G/11074
L2b/UCH1
a.a. position in PmpG (aa 1-600)
D/UW3
E/150
G/11074
L2b/UCH1
A/Har13
1
D/UW3
E/150
G/11074
L2b/UCH1
A/Har13
2
1 → Immunogenicity – Jameson-Wolf
2 → Surface Probability - Emini

D

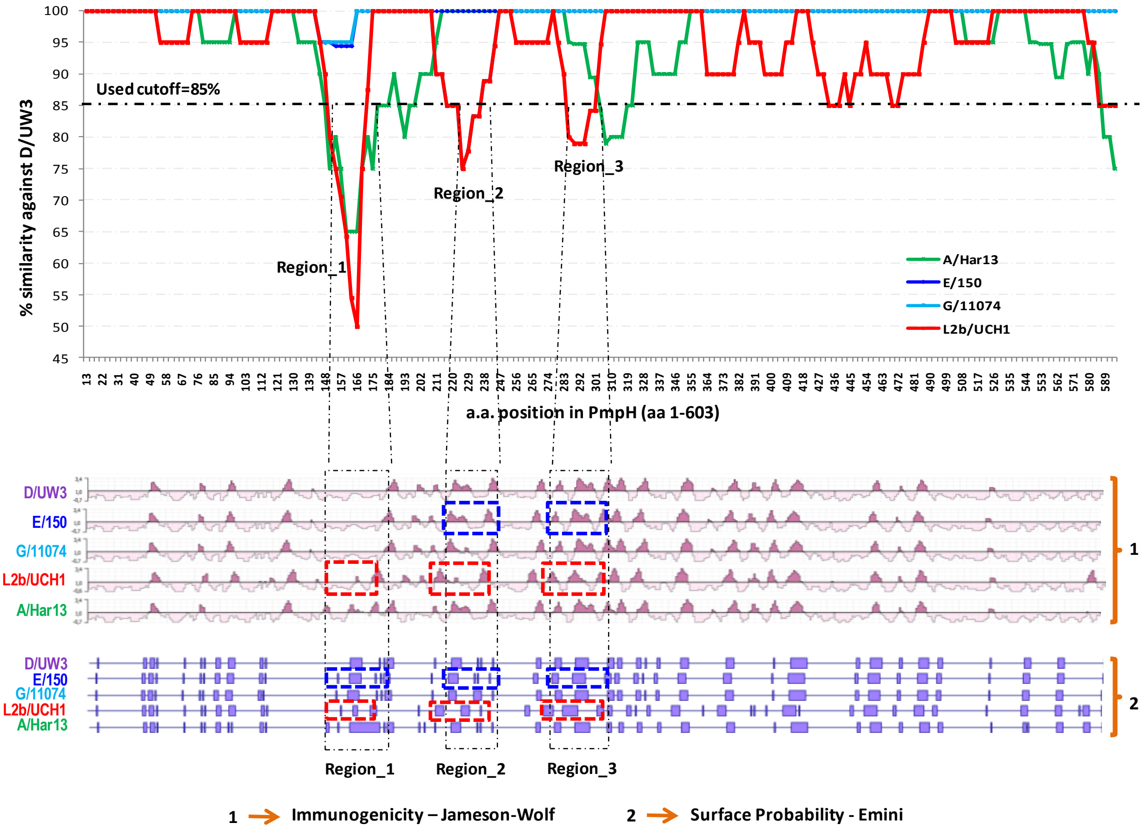

100
95
90
85
80
75
70
65
60
55
50
45
% similarity against D/UW3
Used cutoff=85%
Region_1
Region_2
Region_3
A/Har13
E/150
G/11074
L2b/UCH1
a.a. position in PmpH (aa 1-603)
D/UW3
E/150
G/11074
L2b/UCH1
A/Har13
1
D/UW3
E/150
G/11074
L2b/UCH1
A/Har13
2
Region_1
Region_2
Region_3
1 → Immunogenicity – Jameson-Wolf
2 → Surface Probability - Emini

S3 Fig. Amino acid (a.a) identity throughout the 1st 600 a.a of PmpE (panel A) , PmpF (panel B), PmpG (panel C) and PmpH (panel D) among strains representative of the main branches of each phylogentic tree. All comparisons are made against D/UW3. Higher polymorphic regions (see methods for details) are marked as "Region_#". For each Pmp, the figure also represents both the immunogenicity and surface probability values for each strain under evaluation. The location of all these protein features is adjusted to the horizontal amino acid scale represented in the respective similarity graph.
