# Supplementary material for: Bioinformatic Analysis of Chlamydia trachomatis Polymorphic Membrane Proteins PmpE, PmpF, PmpG and PmpH as Potential Vaccine Antigens
Source: PLoS One. 2015 Jul 1;10(7):e0131695. doi: 10.1371/journal.pone.0131695 (PMC4488443; doi:10.1371/journal.pone.0131695)
Supplement: S1 Table — (PDF) [file pone.0131695.s004.pdf]

S1 Table. *In silico* promoter predictions for *pmpFE* operon regulatory region.

	Promoter sequence	Strain	Score	Software	Comments
P1 [a]	CCCCCACTTAAAGAGCAGCAAGAATAAGCTAGAATCATTGAAAGAAAGAA<br>CCCCCATTTAAAGAGCAGCAAGAATAAGCTAGAATCATTGAAAGAAAGAA	D/UW3<br>E/150<br>E/SW2<br>L2b/UCH1<br>A/Har13	0.80<br>0.98<br>0.98<br>0.98<br>0.98	NNPP<br>NNPP<br>NNPP<br>NNPP<br>NNPP; BPROM	Less probable (too distant from the start codon of *pmpF* and the score obtained for D/UW3 is low).
P2 [b]	GACGTTTGCATCACACAAAAGCTGAGAGATAAAATTAATTACTCCACTTC	D/UW3	0.97	NNPP; BPROM	Most consensual promoter, with a putative A/T spacer and -35 and -10 elements already described in the literature.
	GACGTTTGCATCACACAAAAGCTGAGAGATAAAATTAATTACTCTGCTTC	E/150	0.97	NNPP; BPROM	
	GACGTTTGCATCACACAAAAGCTGAGAGATAAAATTAATTACTCTGCTTC	E/SW2	0.97	NNPP; BPROM	
	GACGTTTGCATCACACAAAAGCTGAGAGATAAAATTAATTACTCCGCTTC	L2b/UCH1	0.97	NNPP	
	GACGTTTGCATCACACAAAAGCTGAAAGATAAAGTTAATTACTCTGCTTC	A/Har13	0.97	NNPP	
P3 [c]	TCCGCTTCATAAAAAATTAATAATTTAATATCAATAAATGTTCTTAT	L2b/UCH1	---	BPROM	Also possible…
P4 [d]	CAATAATTACACTATTTGTAGGCTAAAAATAACATCCCCCTTTTAACCTG	A/Har13	0.81	NNPP	Improbable (low score)

Variable sites among strains are highlighted. Putative -35 and -10 elements are indicated in blue. Putative A/T spacers are underlined. Transcriptional start sites are in red. No transcritptional start sites (TSS) were experimentally identified so far, despite a previous attempt to establish *C. trachomatis* transcriptome (Albrecht et al, 2010).

[a] Despite relatively conserved among all strains, the score obtained for D/UW3 is low (<0.90)…

[b] This is the most consensual σ^{66}-like promoter sequence found among all strains (score=0.97), with a putative A/T spacer that may enhance transcription by RNA polymerase. The -10 element TAAAAT is identical to the -10 hexamer mapped for *ltuB* of both L2/434 and D/UW3 strains (Tan et al, 1998; Fahr et al, 1995; Schaumburg & Tan, 2000) as well as to the -10 hexamer predicted for CT214 of L2/434 (Almeida et al, 2012), for CT249, CT288 and CT646 of D/UW3 (Towsey et al, 2008). The -35 element TTGCAT is identical to -35 hexamer mapped for *hctA* of both L2/434 and D/UW3 strains (Tan et al, 1998; Fahr et al, 1995; Schaumburg & Tan, 2000) and for *clpC* (CT286) of D/UW3 (Towsey et al, 2008) as well as to the -35 hexamer predicted for CT863 of L2/434 (Hefty & Stephens, 2007; Case et al, 2010).

[c] Only predicted for L2b/UCH1. However, the distance between both hexamers is only of 14 nts, which is small when compared to that of all mapped and in silico predicted chlamydial promoters (usually 15-21nts). The -10 element TAATAT is identical to the -10 hexamer predicted for *ssc2* (CT576) of L2/434 strain (Hefty & Stephens, 2007; Case et al, 2010) and to that mapped for *omcA* P1 of D/UW3 (Towsey et al, 2008).

[d] Only predicted for A/Har13, but with a low score (<0.90) and too near of *pmpF* ATG codon.
