# Supplementary material for: Bioinformatic Analysis of Chlamydia trachomatis Polymorphic Membrane Proteins PmpE, PmpF, PmpG and PmpH as Potential Vaccine Antigens
Source: PLoS One. 2015 Jul 1;10(7):e0131695. doi: 10.1371/journal.pone.0131695 (PMC4488443; doi:10.1371/journal.pone.0131695)
Supplement: S2 Table — (PDF) [file pone.0131695.s005.pdf]

S2 Table. *In silico* promoter predictions for *pmpGH* operon regulatory region.

	Promoter sequence	Strain	Score	Software	Comments
P1 [a]	CATTTA**TTGATG**TTAAATTATTAATTTTT**TATGAA**GTGGAGTAATTAATTT	D/UW3	---	BPROM	
	CATTTA**TTGATG**TTAAATTATTAATTTTT**TATGAA**GCAGAGTAATTAATTT	E/150	---	BPROM	Most consensual promoter, with a putative A/T spacer and -35 elements already described in the literature. TSS experimentally identified.
	CATTTA**TTGATG**TTAAATTATTAATTTTT**TATGAA**GCAGAGTAATTAATTT	G/11074	---	BPROM	
	CATTTA**TTGATA**TTAAATTATTAATTTTT**TATGAA**GCGGAGTAATTAATTT	L2b/UCH1	---	BPROM	
	CATTTA**TTGATG**TTAAATCATTAATTTTT**TATGAA**GCAGAGTAATTAACTT [b]	A/Har13	---	---	
P2 [c]	TAATCA**TTGCAG**GTTAAAAGGGGGATGT**TATTTT**TAGCCTGCAAATAGTG	E/150	0.89	NNPP	
		G/11074	0.89	NNPP	Also probable…
	TAATCA**TTGCAG**GTTAAAAGGGGGATGT**TATTTT**TAGCCTACAAATAGTG	A/Har13	0.91	NNPP; BPROM	
P3 [d]	ATTAATTTT**TTATGA**AGTGGAGTAATTAA**TTTTAT**CTCTCAGCTTTTGTG	D/UW3	0.80	NNPP	
	ATTAATTTT**TTATGA**AGCAGAGTAATTAA**TTTTAT**CTCTCAGCTTTTGTG	E/150	0.84	NNPP	Improbable (low scores for all strains except for A/Har13; also too near of *pmpG* ATG codon).
	ATTAATTTT**TTATGA**AGCAGAGTAATTAA**TTTTAT**CTCTCAGCTTTTGTG	G/11074	0.84	NNPP	
	ATTAATTTT**TTATGA**AGCGGAGTAATTAA**TTTTAT**CTCTCAGCTTTTGTG	L2b/UCH1	0.82	NNPP	
	ATTAATTTT**TTATGA**AGCAGAGTAATTAA**CTTTAT**CTTTCAGCTTTTGTG	A/Har13	1.00	NNPP	
P4	TAGCTTGCAAAATGGATATAGTTGAAAGATAAAAAAAACTGAGGCAAGCA	D/UW3	0.89	NNPP	Improbable (only for some genital strains with low scores; also too distant from *pmpG* ATG codon).
		L2b/UCH1	0.89	NNPP	
P5 [e]	AATTA**TTGGAT**TTTGTAAAGAGAGAAGGA**TCGAAT**GCGCCGAAGATAAGG	E/150	0.99	NNPP	
		G/11074	0.99	NNPP	Also probable…
		A/Har13	0.99	NNPP	

Variable sites among strains are highlighted. Putative -35 and -10 elements are indicated in blue. Putative A/T spacers are underlined. Transcriptional start sites are in red. The TSS experimentally found by Albrecht at al (2010) is also highlighted in blue.

[a] This is the most consensual σ^{66}-like promoter sequence found among all strains, with a putative A/T spacer that may enhance transcription by RNA polymerase. The -35 element TTGATA is identical to -35 hexamer mapped for *omcA* P1, *crpAB* P2, CT602 and *tuf* (CT322) P1 of L2/434, F or D/UW3 strains (Tan et al, 1998; Fahr et al, 1995; Shen et al, 2000; Schaumburg & Tan, 2000; Towsey et al, 2008) as well as to the -35 hexamer already predicted for CT035, CT249 and CT254 of D/UW3 (Towsey et al, 2008). The -35 element TTGATG is identical to that also predicted for CT602 and *rpoC* (CT314) of D/UW3 strain (Towsey et al, 2008).

[b] The existence of this putative promoter on A/Har13 was not supported by any in silico prediction.

[c] Putative σ^{66}-like promoter sequence alternatively found solely for A/Har13 strain and some genital strains, with a putative A/T spacer. No homology was found with any -35 or -10 element already described in the literature.

[d] The -10 element TTTTAT is identical to the -10 hexamer predited for CT255 and *hctA* (CT743) (Towsey et al, 2008). The -35 element TTATGA is identical to that mapped for *lutB* of L2/434 strain (Tan et al, 1998; Fahr et al, 1995).

[e] No homology was found with any -35 or -10 element already described in the literature.
